# 3D Multilayered DDM-Modified Nickel Foam Electrode for Advanced Alkaline Water Electrolysis

**DOI:** 10.3390/molecules31010069

**Published:** 2025-12-24

**Authors:** Elitsa Petkucheva, Galin Borisov, Jordan Iliev, Elefteria Lefterova, Evelina Slavcheva

**Affiliations:** 1 Acad. Evgeni Budevski Institute of Electrochemistry and Energy Systems Bulgarian Academy of Sciences (IEES-BAS), Acad. G. Bonchev bl. 10, 1113 Sofia, Bulgaria; gal.rusev@iees.bas.bg (G.B.); joiliev@iees.bas.bg (J.I.); e.lefterova@iees.bas.bg (E.L.); eslavcheva@iees.bas.bg (E.S.); 2 Center of Competence “HITMOBIL”, (CoC), Acad. G. Bonchev Str. 10, 1113 Sofia, Bulgaria

**Keywords:** alkaline water electrolysis, zero-gap cell, nickel foam, dip and drying method

## Abstract

Advanced alkaline water electrolysis (AWE) in “zero-gap” configuration is a promising approach for low-temperature hydrogen production, but its efficiency strongly depends on the design and surface chemistry of nickel-based electrodes. Here, we present a simple dip-and-drying method (DDM) to modify commercial nickel foam with a Ni–FeOOH/PTFE microporous catalytic layer and evaluate its electrochemical performance in 1 M KOH and in a laboratory zero-gap cell with a Zirfon^®^ Perl 500 UTP diaphragm, through circulating 25 wt.% KOH. The FeSO_4_-assisted DDM treatment generates mixed Ni–Fe oxyhydroxide surface species, while PTFE imparts control hydrophobicity, enhancing both catalytic activity and gas-release behavior. Annealing the electrode (DDM-NF-CAT-A) results in a cell voltage of 2.45 V at 1 A·cm^−2^ and 80 °C, demonstrating moderate performance comparable to other Ni-based electrodes prepared via low-complexity methods, though below that of optimized state-of-the-art zero-gap systems. Short-term durability tests (80 h at 0.5 A·cm^−2^) indicate stable operation, but long-term industrial performance was not assessed. These findings illustrate the potential of the DDM approach as a simple, low-cost route to structured nickel foam electrodes and provide a foundation for further optimization of catalyst loading, microstructure, and long-term stability for practical AWE applications.

## 1. Introduction

Alkaline water electrolysis (AWE) is one of the most established and industrially deployed technologies for large-scale hydrogen production, offering long operational history, low system cost, and the ability to use earth-abundant materials as electrodes [1,2,3,4,5]. Water splitting in alkaline media proceeds through the oxygen evolution reaction (OER) at the anode and the hydrogen evolution reaction (HER) at the cathode:

At anode:     4OH^−^ → O_2_ + 2H_2_O + 4e^−^ (1)

At cathode:     H_2_O + 4e^−^ → H_2_ + 2OH^−^ (2)

Overall:     2H_2_O → 2H_2_ + O_2_ (3)

Industrial electrolyzers typically operate in concentrated KOH or NaOH solutions (25–30 wt.%) and rely on porous nickel-based electrodes—such as Raney Ni or Ni foams—owing to their catalytic activity, durability, and effective gas removal [6,7,8,9]. Product gases are separated by a hydroxide-conductive, gas-impermeable diaphragm that minimizes crossover while maintaining ionic transport [10,11]. Despite these advantages, conventional alkaline cells still face challenges, including carbonate accumulation, electrode degradation, and efficiency losses at high current densities, motivating the development of advanced electrode materials, improved diaphragms, and optimized cell architectures.

Over the past decade, major progress has been made in designing highly active electrocatalysts for both OER and HER, particularly those based on transition metals such as Ni, Fe, Co, Mo, and W [12,13,14,15,16,17,18,19,20,21]. Ni-, Co-, and Fe-based oxyhydroxides and their derivatives (LDHs, oxides, chalcogenides, and pnictides) have demonstrated excellent alkaline OER activity by enabling favorable redox transitions and enhanced charge transport [18,19,22,23,24,25,26,27,28,29,30,31,32]. Correspondingly, porous Ni foams and meshes remain preferred substrates due to their low cost, mechanical robustness, and compatibility with large-scale manufacturing [33,34,35,36,37,38,39,40,41,42,43]. Parallel improvements in diaphragm technologies—most notably Zirfon^®^—have yielded separators with exceptional chemical stability, hydrophilicity, and mechanical strength in 30 wt.% KOH at elevated temperatures [5,44,45,46,47].

The great progress has been achieved not only in electrocatalysis, separators/membranes and cell design, but also in the related fields such as process optimization and system modeling [9,34,41,42,43,48,49,50,51,52,53].

A particularly significant advancement in recent years is the implementation of zero-gap electrolyzer configurations, where electrodes are pressed directly against the separator. This arrangement minimizes ohmic resistance, eliminates electrolyte-filled gaps, and enhances mass transport [54,55,56]. Zero-gap systems integrating Zirfon^®^ diaphragms have demonstrated substantial reductions in voltage losses and robust high-current-density operation [51,55,57,58,59,60,61]. Representative examples include NiAlMo/Ni achieving 1.90 V at 1 A cm^−2^ and 80 °C [51], NiFe-GDE/Ni delivering ~2.0 V at 0.4 A cm^−2^ [62], and sputtered Ni-Mo systems operating at 1.71 V at 100 mA cm^−2^ [63]. These studies highlight the critical role of electrode microstructure, wettability, and catalyst composition in zero-gap performance.

However, traditional methods for modifying Ni-based electrodes—such as electrodeposition, hydrothermal growth, chemical oxidation, and PVD sputtering—often involve high temperatures, long processing times, costly precursors, or complex equipment [64,65,66,67,68,69,70,71,72,73]. While they can deliver high catalytic activity, many approaches neglect the equally important role of bubble dynamics and pore wetting behavior in zero-gap environments, where gas blockage can dominate losses at current densities above 0.5–1 A cm^−2^.

This motivates the pursuit of simple, scalable surface-engineering strategies that enhance both catalytic properties and gas–liquid management. The dip-and-drying method (DDM) has recently emerged as a low-cost, solution-based coating technique capable of forming homogeneous layers on 3D porous substrates without the need for high-temperature processing or specialized equipment [74,75,76]. Unlike chemical growth methods, DDM relies on physical deposition from a suspension, enabling straightforward scalability.

In this work, we introduce a DDM-modified Ni foam electrode (DDM-NF-CAT) incorporating Fe-containing precursors and a PTFE binder to create a hierarchical, dual-function surface. Upon drying and electrochemical activation, the Fe species convert into mixed Ni–Fe oxyhydroxides—well-known high-activity OER precatalysts—while PTFE forms hydrophobic microdomains that facilitate bubble detachment and gas–liquid separation during high-rate operation. This unique combination of hydrophilic catalytic sites and hydrophobic escape channels produces a functional architecture that is difficult to achieve via electrodeposition or hydrothermal synthesis. The approach thus aligns catalytic enhancement with improved gas-release behavior—two key requirements for zero-gap AWE.

The DDM-NF-CAT electrode is integrated as the anode in a laboratory-built zero-gap electrolyzer featuring a Zirfon^®^ PERL 500 separator, circulating 25 wt.% KOH electrolyte, and an acid-washed Ni foam cathode. The cell delivers 2.2 V at 0.140 A cm^−2^ and 80 °C under quasi-steady-state operation. After thermal annealing, the DDM-NF-CAT-A electrode demonstrates improved performance and short-term stability, achieving 2.45 V at 1 A cm^−2^—values competitive with other Zirfon^®^-based zero-gap systems (Table 1), despite the simplicity and scalability of the fabrication process. These results underscore the practicality of the DDM approach while clarifying that its primary contribution lies in offering a low-complexity pathway for manufacturing functional electrodes for next-generation alkaline water electrolysis.

## 2. Results and Discussion

### 2.1. Physical Characterization Analysis

The textural properties (incl. surface area, total pore value, and pore size) of pristine Ni powder and DDM-functionalized Ni powder (DDM-Ni-P where P is devoted to powder) were evaluated via N_2_ adsorption/desorption isotherms at 77 K, and analyzed using the Brunauer–Emmett–Teller (BET) and Barrett–Joyner–Halenda (BJH) methods. The results, summarized in Table 2, include the specific surface area (m^2^·g^−1^), total pore volume (cm^3^·g^−1^), and average pore diameter (nm), with uncertainties representing the standard deviation from triplicate measurements *(n* = 3).

The BET analysis reveals that DDM treatment induces a modest increase in specific surface area, from 1.3 ± 0.05 m^2^·g^−1^ for pristine Ni to 1.6 ± 0.05 m^2^·g^−1^ for DDM-Ni-P, indicating a slight enhancement of the accessible surface. The total pore volume remains essentially unchanged at 0.06 cm^3^·g^−1^, suggesting that the overall porosity is not substantially affected by the surface modification.

Examination of pore size distribution shows that the adsorption branch BJH diameter remains constant at 2.1 nm, whereas the desorption branch diameter decreases from 2.2 nm for pristine Ni to 1.7 nm after DDM treatment. Consequently, the average pore diameter decreases from 18 nm to 16 nm. This reduction in pore size, in the context of a nearly constant total pore volume, implies that DDM treatment promotes the formation of smaller, more uniform pores, thereby increasing the effective surface area without significantly altering bulk porosity.

The BET and BJH results indicate that DDM functionalization subtly enhances surface area while decreasing average pore diameter, potentially improving surface accessibility and reactivity for catalytic or electrochemical applications.

**Figure 1:**
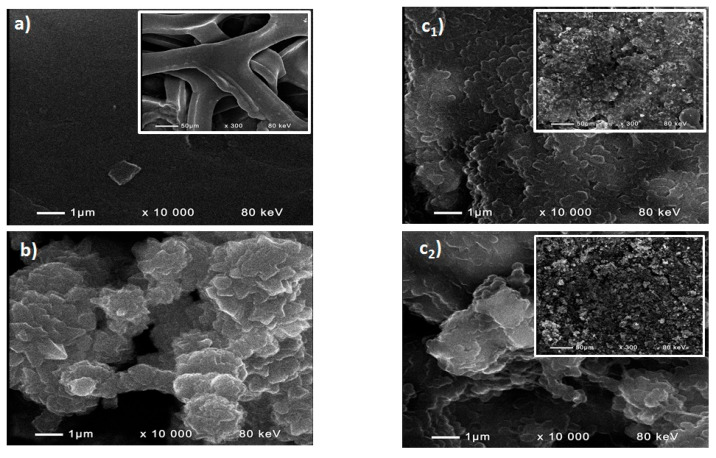
SEM images of (**a**) NF-AW; (**b**) DDM-Ni-P; (**c_1_**) DDM-NF-CAT; (**c_2_**) DDM-NF-CAT-A.

The scanning electron microscopy (SEM) images are presented in Figure 1. It is seen that the acid-washed nickel foam (NF-AW) has a highly developed surface area (Figure 1a). The DDM-Ni-P (Figure 1b) form mesostructured and homogeneously distributed particles with sizes of 100–300 nm. The catalyst particles are evenly distributed on both electrodes. The annealing of the DDM-NF-CAT (Figure 1c_1_) leads to aggregation of the catalyst particles and the formation of cracks on the electrode surface well, which are visible on the inset in Figure 1c_2_.

**Figure 2:**
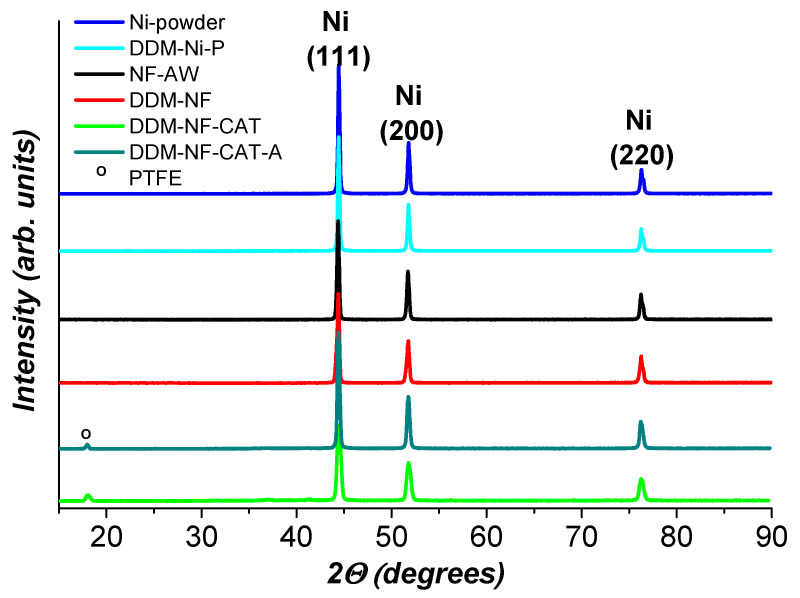
XRD patterns of the samples under study.

**Figure 3:**
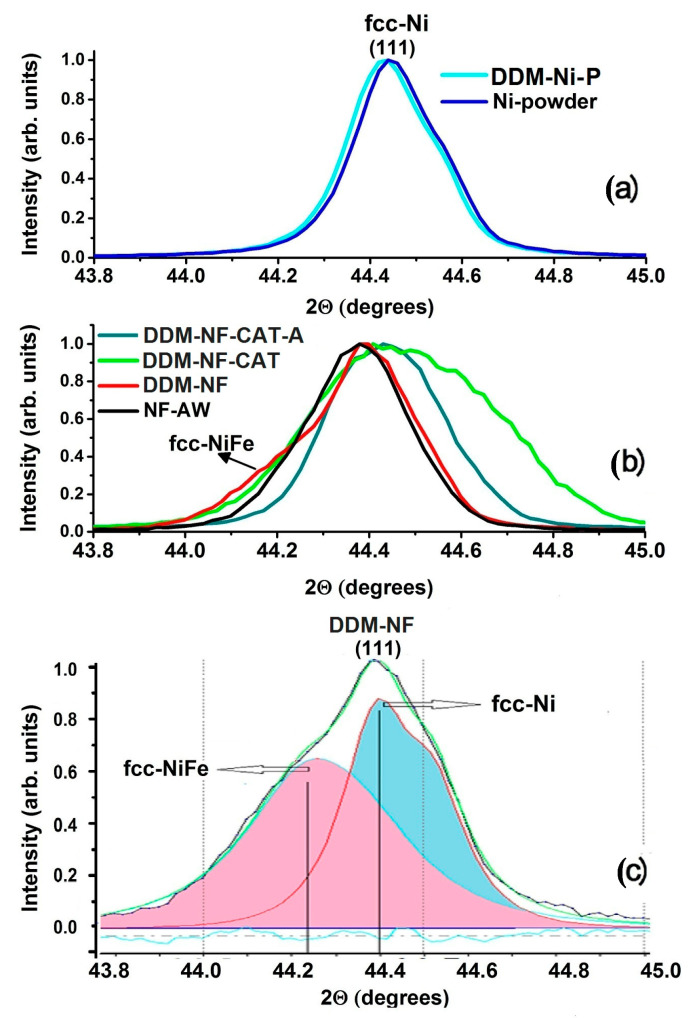
Normalized Ni (111) XRD peaks of the samples under study (**a**) Ni -powders catalysts, (**b**) electrodes and (**c**) zoom of the fcc-NiFe peak of the DDM-NF electrode where: the black dot curve is the experimentally obtained, the green dashed curve is the fitted experimental curve; the pink and the blue sections are deconvoluted peaks and the dashed blue line is a baseline.

The X-ray phase analysis data presented in Figure 2 shows only the presence of fcc-Ni in the patterns of all samples tested. Neither iron nor its oxides are registered. PTFE is seen for DDM-NF-CAT. The XRD patterns of Ni-powder and DDM-Ni-P are identical and the peak positions correspond to pure fcc-Ni (Figure 3a). The FWHM is 0.55° and the corresponding crystallite size is 73 nm.

A shoulder on the smaller angle side on the (111) peak of the DDM-NF sample appears (Figure 3b). It was fitted with two components—one corresponding to fcc-Ni (peak at 2θ = 44.96°), and the other (2θ = 44.228°) (probably related to a thin fcc-NiFe layer formed on the NF surface (Figure 3c). The ratio of the intensities is 56.55%:43.5%, respectively. The strong widening (FWHM = 0.411°) of the DDM-NF-CAT diffraction peaks is due to small crystallite size (21 nm). After annealing, the peak narrows to 0.245° because of the increase in crystallite size (~38 nm).

**Figure 4:**
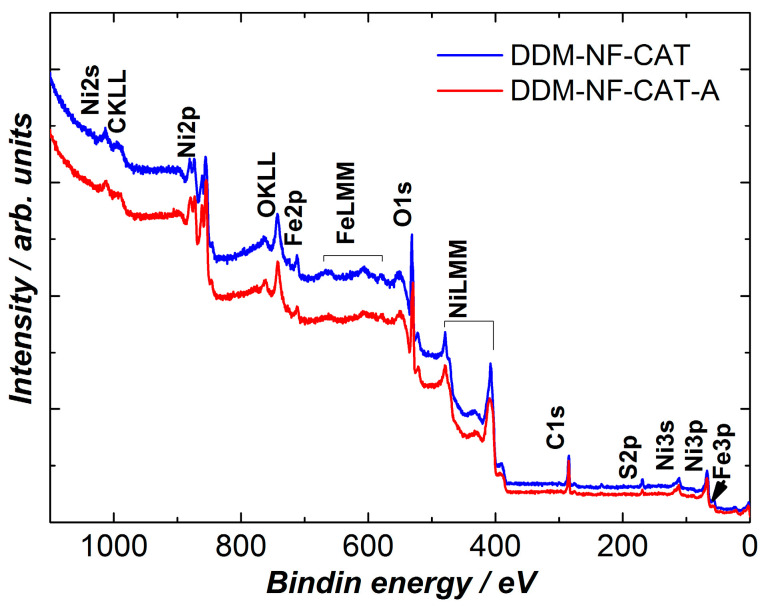
Survey XPS spectra of the electrodes before and after electrode annealing.

The iron presence in the catalyst was checked by X-ray photoelectron spectroscopy (XPS). The survey spectra of the samples DDM-NF-CAT are presented in Figure 4.

**Figure 5:**
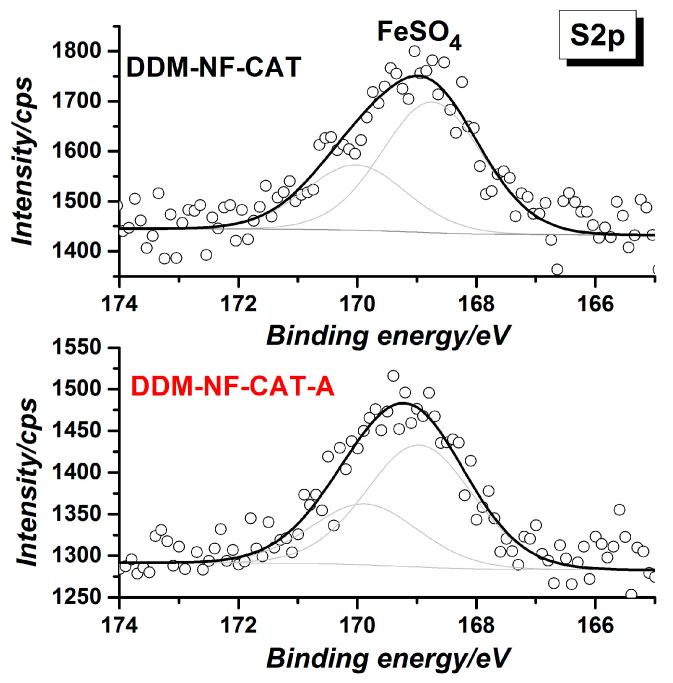
S2p high-resolution XPS spectra of the electrodes before and after electrode annealing. The black dotted curves are the experimentally obtained data, the bold black curves are the fitted experimental curves and the inset curves are the deconvoluted peaks.

**Figure 6:**
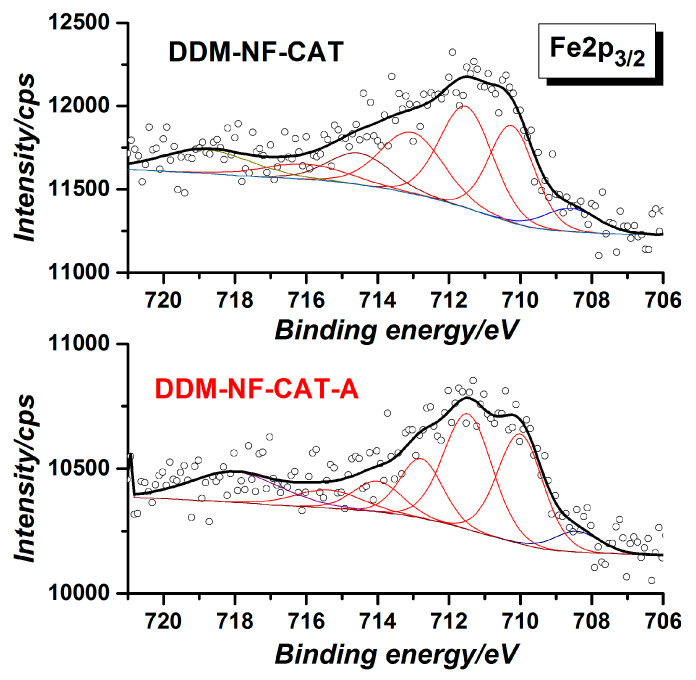
Fe2p_3/2_ high-resolution XPS spectra of the electrode before and after annealing. The black dotted/hollow circles curves are the experimentally obtained data, the bold black curves are the fitted experimental curves and the inset curves are the deconvoluted peaks.

**Figure 7:**
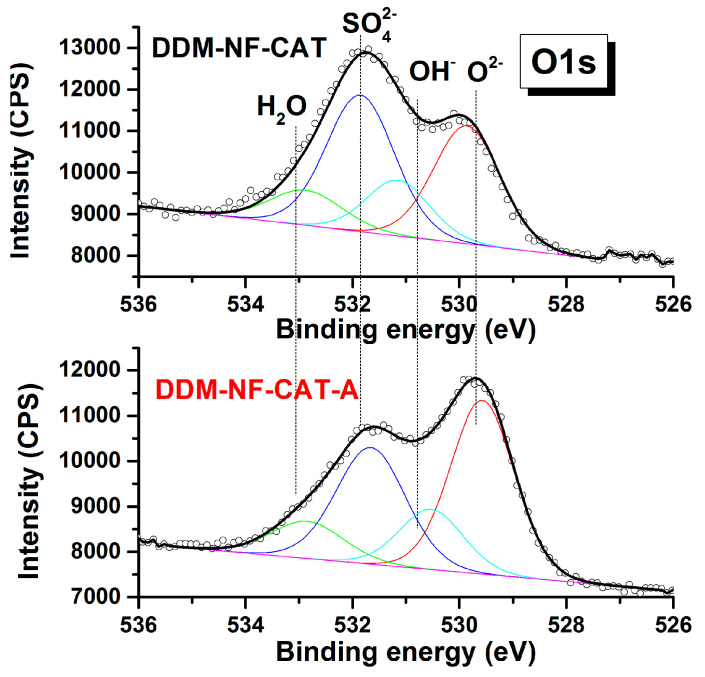
O1s high-resolution XPS spectra of the electrodes before and after annealing. The black dotted curves/hollow circles are the experimentally obtained data, the bold black curves are the fitted experimental curves and the inset curves are the deconvoluted peaks.

Besides nickel and carbon, the survey of XPS spectrum shows the presence of Fe, S, and O (Table 3). The presence of FeSO_4_, as well as of other oxides, is confirmed by S2p, Fe2p, and O1s’ high-resolution spectra (Figure 5, Figure 6 and Figure 7). S2p binding energy at ~169 eV (Figure 5) is attributed to the sulfate group in FeSO_4_ [80]. The Fe2p3/2 high-resolution spectrum corresponds to Fe(II) in FeSO_4_ [81].

**Figure 8:**
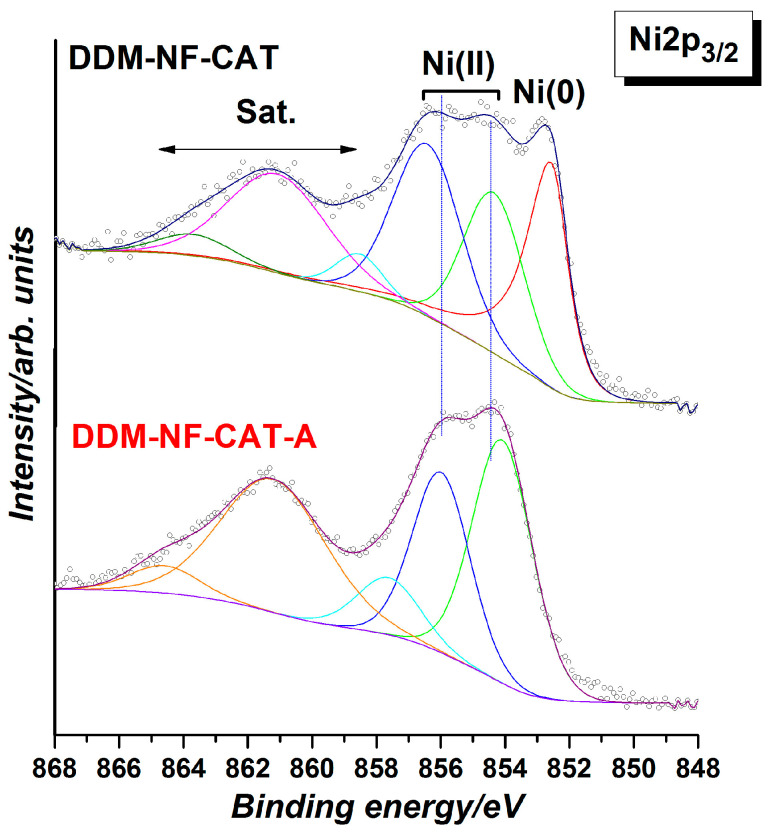
High-resolution Ni2p_3/2_ XPS spectra of the electrodes before and after annealing. The black dotted curves/hollow circles are the experimentally obtained data, the bold curves are the fitted experimental curves and the inset curves are the deconvoluted peaks.

The weak satellite at ~719 eV indicates the presence of Fe(III) [82,83]. The O1s peak shows different oxygen species. According to [68,69], the main two components at ~531.7 and ~529.7 eV are due to SO_4_^−^ and O^2−^ in metal oxides (MOs), respectively. In the high-resolution Ni2p spectrum shown in Figure 8, the MO corresponds to Ni(II). The component at ~854 eV together with 856 V and the corresponding satellites are characteristic of NiO [84]. This means that part of the Ni surface is oxidized. After the thermal treatment metal component Ni(0) at (852 eV) disappears, i.e., the Ni surface is fully oxidized, the amount of NiO on the surface increases by about 4 at.%, mainly at the expense of Fe and S. This correlates with the increase in O^2−^ oxygen component and the decrease in SO_4_^−^ component in the O1s HR spectra (Figure 7).

**Figure 9:**
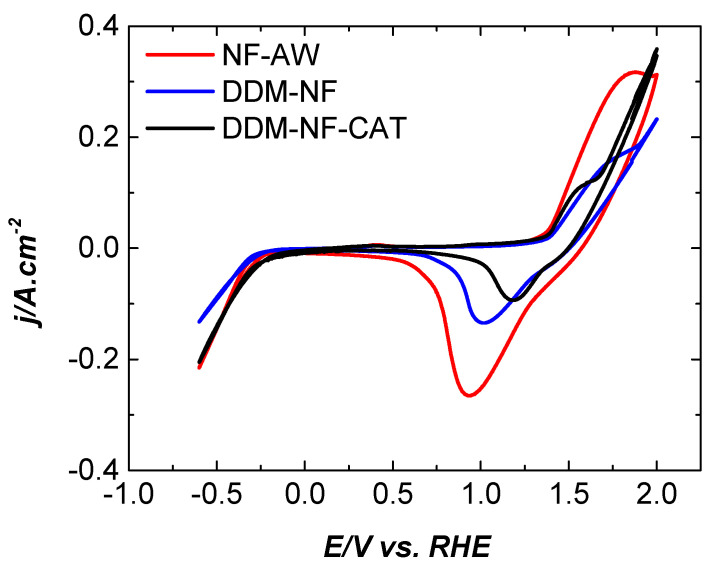
Cyclic voltammograms of the electrodes in 1 M KOH, with a scan rate of 100 mV·s^−1^.

The cyclic voltammograms of the electrodes under study are compared in Figure 9. The reduction peak potential shifts in a positive direction in the following order: E_red_(tNF-AW) > E_red_(DDM-tNF) > E_red_(DDM-tNF-Cat.). A shift in oxidation peak potential is not observed, while a decrease in the onset potential for OER is observed for the DDM-NF-CAT.

**Figure 10:**
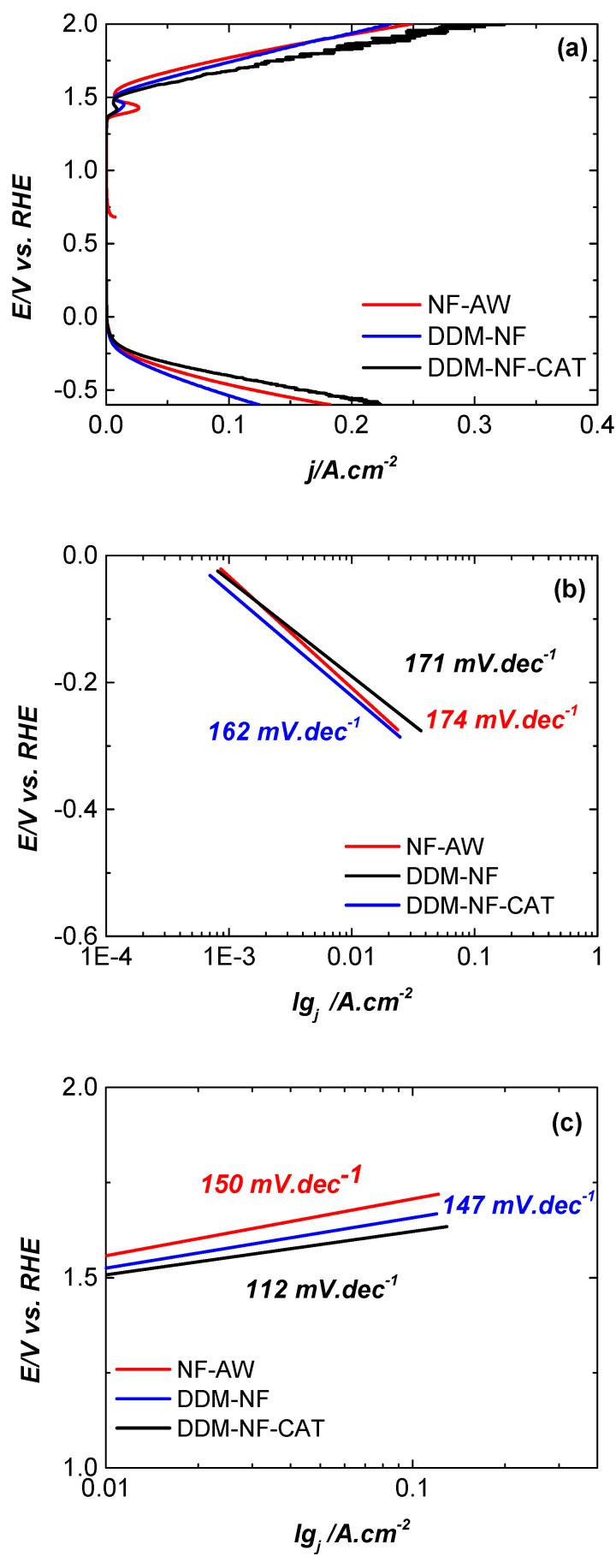
(**a**) Anodic and cathodic polarization curves of the electrodes under study in 1 M KOH, with a scan rate 1 mV·s^−1^, (**b**) HER Tafel slopes, and (**c**) OER Tafel slopes.

The steady-state polarization data of the HER (Figure 10a) show that the application of the catalytic layer (DDM-NF-CAT) improves the activity towards HER with an overpotential of 190 mV at 10 mA cm^−2^ compared to the pure NF-WA cathode. The calculated Tafel slope (Figure 10b) is 162 mV·dec^−1^. The performance of this electrode in 1 M KOH electrolyte is comparable to bare nickel foam hydrogen electrodes [85]. This value departs from the canonical 120 mV·dec^−1^ often associated with a single elementary Volmer step on ideal, planar electrodes. However, Tafel slopes measured on porous, three-dimensional electrodes—particularly when uncompensated for ohmic drop—reflect a convolution of intrinsic charge-transfer kinetics, mass-transport limitations, double-layer charging, and geometric effects (porosity, roughness, and bubble dynamics). Therefore, the observed HER slope should be interpreted cautiously and not taken alone as definitive mechanistic proof [86].

The OER activity of DDM-NF-CAT also improved relative to bare NF-AW: the electrode displays an overpotential of ≈270 mV at 10 mA·cm^−2^ (Figure 10a) and reaches ≈300 mA·cm^−2^ at a cell potential of 2.0 V in our zero-gap arrangement. The OER Tafel slope obtained from the linear region described above is ≈112 mV·dec^−1^. We emphasize that assigning a single elementary step (e.g., “Volmer”) to this slope is not appropriate for OER in alkaline media; the OER mechanism on Ni-based electrodes typically involves multiple proton-coupled electron-transfer and adsorbate–evolution steps and can be strongly influenced by surface oxidation state, coverage of oxyhydroxide species, and transport effects [87,88]. In our case, the measured OER slope likely reflects mixed control (kinetic + transport + ohmic) under uncompensated conditions. Definitive mechanistic interpretation would require iR-corrected polarization, operando spectroscopic probes of surface speciation, and EIS to separate kinetic and mass-transport resistances.

### 2.2. Electrode Performance in “Zero-Gap” Electrolysis Cell

The experiments are caried out with two CATHODE | Zirfon^®^ Perl 500 UTP | ANODE cell configurations, namely NF-AW | Zirfon^®^ Perl 500 UTP | DDM-NF-CAT and Ni-AW | Zirfon^®^ Perl 500 UTP | DDM-NF-CAT-A.

**Figure 11:**
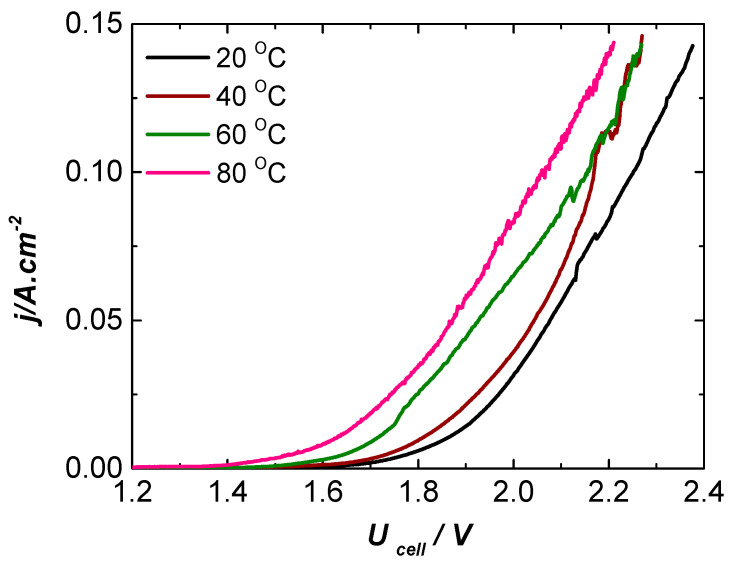
Current density–cell voltage characteristics of NF-AW| Zirfon^®^ Perl 500 UTP | DDM-NF-CAT in the temperature range of 20–80 °C.

Figure 11 shows the current density–cell voltage characteristics for the NF-AW | Zirfon^®^ Perl 500 UTP | DDM-NF-CAT configuration operating at different temperatures, typical for a variety of industrial alkaline electrolysis applications. The current density reached at 80 °C and cell voltage of 2.2 V is 0.140 A·cm^−2^, with no indication of electrode degradation.

**Figure 12:**
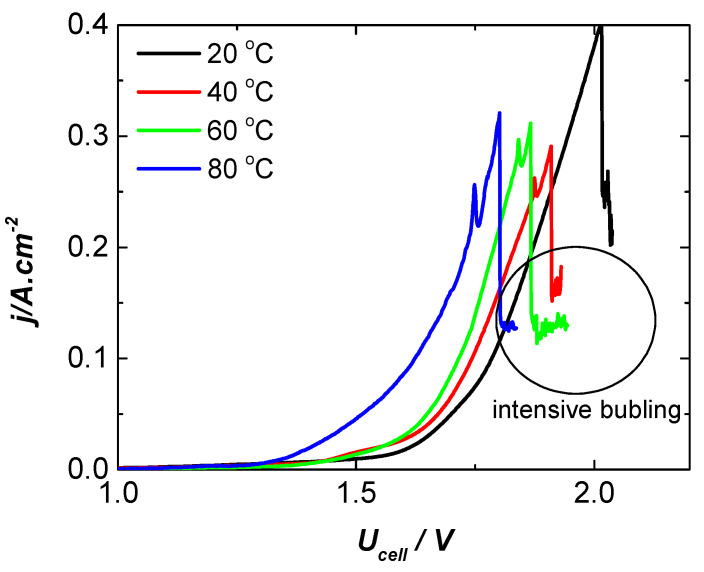
Current density–cell voltage characteristics of NF | Zirfon^®^ Perl 500 UTP | DDM-NF- CAT-A in the temperature range of 20–80 °C.

Figure 12 presents the performance of the cell configuration NF-AW | Zirfon^®^ Perl 500 UTP | DDM-NF-CAT-A at the same operating conditions. Generally, the gas evolution rate is higher at elevated temperatures due to higher electrode activity and faster ion transport (regardless of the electrode type used). However, the rapid gas generation on the electrode surface does not guarantee a quantitative increase in the amount of gas emitted from the electrolysis cell due to collecting of the gas bubbles on the electrode surface.

The polarization curves recorded at 20, 40, 60, and 80 °C in the zero-gap cell exhibit the expected strong temperature dependence of alkaline water electrolysis, consistent with prior observations for Zirfon^®^-based zero-gap systems [89,90,91,92].

Increasing temperature simultaneously accelerates both the HER and OER, lowering the cell voltage at a given current density due to the enhanced ionic conductivity of concentrated KOH and reduced ohmic resistance [93,94], as well as faster charge-transfer kinetics [95], and decreased electrolyte viscosity that promotes bubble detachment and mass transport at the electrode/diaphragm interface [96]. The region marked as “intensive bubbling” corresponds to the onset of vigorous O_2_ evolution, where bubble coalescence and transient gas accumulation locally distort electrolyte thickness and generate characteristic current–density fluctuations; such behavior is well-documented for hydrophobicity-modified Ni and Ni–Fe electrodes under high-rate alkaline conditions [97,98]. Overall, the progressively improved activity observed with increasing temperature aligns with established descriptions of bubble-regulated transport limitations in zero-gap alkaline electrolysis.

**Figure 13:**
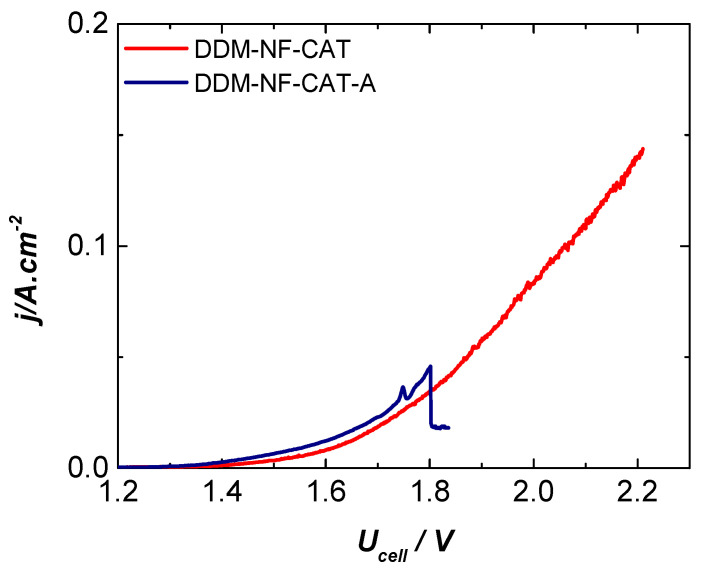
Comparison of the current density–cell voltage characteristics of the cells with annealed (DDM-NF-CAT-A) and non-annealed (DDM-NF-CAT) anodes at 80 °C.

During electrolysis, a vigorous gas evolution is observed on both anode and cathode sides. The excessive gas generation on the NF-AW | Zirfon^®^ Perl 500 UTP | DDM-NF-CAT-A cell configuration is visible in Figure 12 and Figure 13 and leads to shielding of the electrode surface (blocking the electroactive surface sites). The annealed electrode shows lower overvoltage compared to the assembly with the non-annealed electrode. The enhanced OER performance of DDM-NF-CAT-A can be attributed to two synergistic effects arising from mild annealing at 340 °C: (i) modification of surface chemistry that promotes the formation of mixed Ni–Fe (oxy)hydroxide domains, and (ii) annealing-induced microstructural changes that improve gas removal. The thermal treatment partially oxidizes the near-surface Ni lattice and enables Fe diffusion, resulting in Ni–Fe (oxy)hydroxide phases that exhibit intrinsically superior OER activity—15–30× higher than pure NiOOH [87,88] and a reduction in charge-transfer resistance due to an improved short-range [99]. In parallel, SEM analysis reveals microcracks and surface texturing consistent with partial PTFE reorganization, generating hydrophobic microdomains that lower bubble adhesion, accelerate bubble detachment, and enhance electrolyte renewal [92,97]. These morphological effects collectively shorten bubble residence time, prevent active-site blockage, and improve mass transport, particularly in zero-gap architectures where gas accumulation strongly affects cell voltage. The noisy voltage oscillations observed at high current density further support a bubble-regulated regime dominated by intermittent accumulation and detachment. While these interpretations align with established behavior of Ni–Fe alkaline anodes and PTFE-modified porous electrodes, the mechanism remains a hypothesis that requires operando validation through high-resolution SEM, bubble-dynamics imaging, and EIS-based mass-transport analysis.

**Figure 14:**
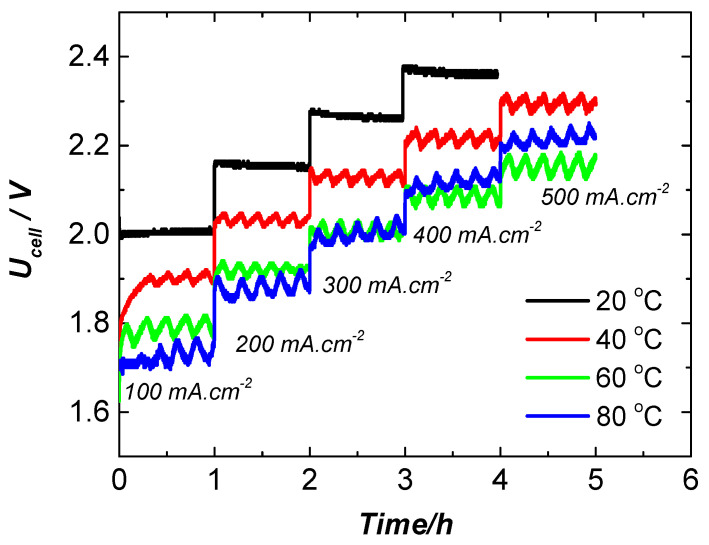
Galvanostatic polarization tests of NF-AW | Zirfon^®^ Perl 500 UTP | DDM-NF-CAT-A at a temperature range of 20–80 °C (step 20 °C) and current density range of 0.1–0.5 A·cm^−2^ (step 0.1 A·cm^−2^).

The electrochemical performance of the electrolyzer is explored by galvanostatic polarization applying constant current in the range of 0.100–0.500 A cm^−2^ (Figure 14). The experiment is carried out stepwise with a current density increment of 100 mA cm^−2^ and step duration of 60 min at four selected temperatures—20 °C, 40 °C, 60 °C, and 80 °C.

**Figure 15:**
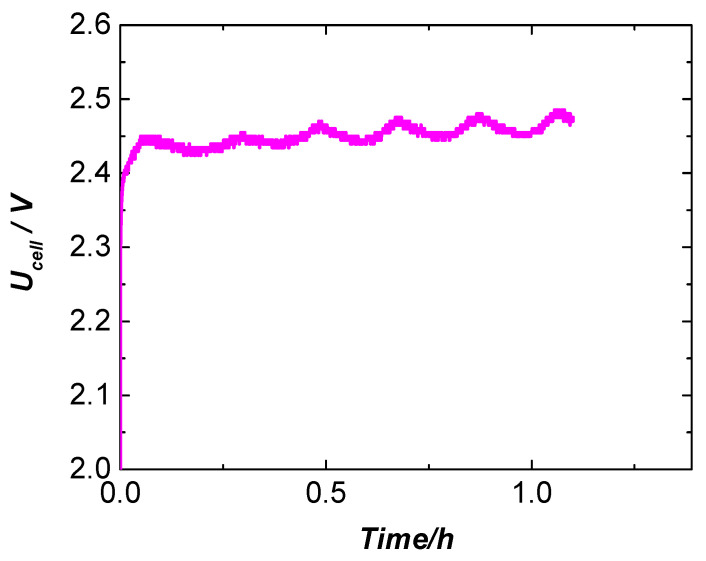
Constant current operation of the “zero-gap” electrolysis cell with NF-AW | Zirfon^®^ Perl 500 UTP | DDM-NF-CAT-A configuration at 1 A cm^−2^, 80 °C, and time of 1 h.

Figure 15 shows the galvanostatic polarization curve recorded at a current density of 1 A·cm^−2^ at 80 °C of the cell with a DDM-NF-CAT-A anode. The cell voltage reaches a value of only 2.45 V, which sustains for the whole test duration. Table 4 summarizes the obtained values of cell voltage and cell power at various constant current densities. The achieved maximum power density of the cell with DDM-NF-CAT-A anode at 80 °C is 2.45 W cm^–2^ (or W_cell_ = 17.15 W) at a current density of 1 A cm^−2^.

The electrochemical stability of the cell is further examined under long-term galvanostatic “on–off” operation at room temperature. The constant current of 500 mA cm^−2^ is applied on the cell for 10 h; then, it is switched off and the open-circuit potential is followed for another 10 h. This “on–off” operation is repeated for 80 h.

**Figure 16:**
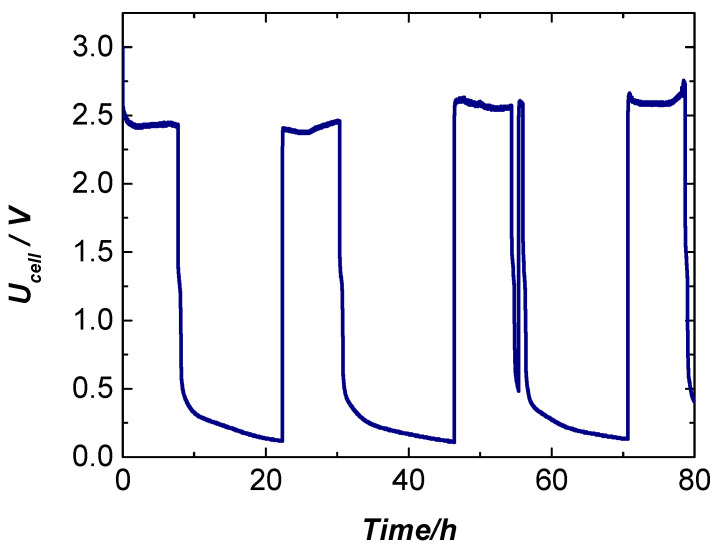
Durability “on–off” test: Applied current density is 0.5 A·cm^2^ OCP, each with duration of 10 h at 20 °C.

The results presented in Figure 16 show that during the “on” phase of each “on–off cycle”, the cell voltage rapidly stabilizes at approximately 2.5 V, indicating fast activation of the electrode surface and immediate establishment of steady-state operation. In contrast, upon switching off the current, the voltage decays more slowly toward the open-circuit potential (OCP), reflecting relaxation processes associated with double-layer discharge and redistribution of reaction intermediates at the electrode interface. Notably, during the third on–off cycle, a slight increase in the steady-state voltage to 2.73 V is observed, suggesting partial blocking of surface-active sites, likely due to adsorption of intermediate species or localized surface restructuring. This effect appears to be reversible, as no structural degradation of the cell or the electrodes is detected over the duration of the experiment, implying that the active material maintains its intrinsic stability under repeated cycling. The observed behavior highlights the dynamic nature of the electrode–electrolyte interface, emphasizing the importance of surface accessibility and site availability for sustained electrochemical performance. These findings provide valuable insight into the short-term operational stability of the system and suggest that the material is capable of maintaining functional integrity under intermittent current conditions, which is relevant for practical electrolysis applications where start–stop operation is common.

## 3. Materials and Methods

### 3.1. Electrode Preparation

**Figure 17:**
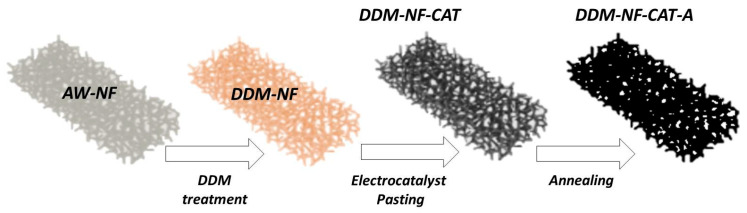
Scheme of the electrode preparation procedure.

The electrodes under study are cut from a commercial sheet of Nickel foam (NF) (Xiamen Tmax Battery Equipment Limited, Xiamen, China) with the following specifications: thickness 1 mm; surface density 0.346 g/m^2^; porosity ≥ 95% (80–110 pores per inch, average hole diameters about 0.25 mm); purity > 99.8% (excellent anti-corrosive). The all used chemicals were from Sigma Aldrich (St. Louis, MO, USA). The NF electrodes are rinsed several times with acetone to remove grease, surface, and pore contamination and then dipped in 10% HCl for 30 min in the sonificator at room temperature to remove surface oxides and to increase the active surface area (denoted NF-AW, acid-washed) [66]. After cleaning, the electrodes are catalyzed, applying a four-step procedure illustrated schematically in Figure 17.

Step 1: Perform treatment of Ni-powder in 0.01 M aqueous solution of FeSO_4_ for 15 min and dry in air atmosphere overnight (the samples are denoted as DDM-Ni-P).

Step 2: Prepare catalytic ink by mixing DDM-Ni-P with 10 wt.% PTFE emulsion and ethanol (the samples are denoted as CAT).

Step 3: Perform nickel foam (NF) treatment via ‘’DDM’’—dipping of NF-AW samples in 0.01 M aqueous solution of FeSO_4_ for 15 min and drying in air atmosphere overnight (the samples are denoted as DDM-NF).

Step 4: Paste the CAT on DDM-NF (the samples are denoted as DDM-NF-CAT).

For further optimization, the DDM-NF-CAT electrodes are annealed at 340 °C (heating rate: 2 °C min^−1^), followed by a 30 min hold (denoted as DDM-NF-CAT-A).

The areal catalyst loading was determined gravimetrically from the mass difference before and after deposition and normalized to the geometric electrode area. The loading amounted to 1.25 ± 0.05 mg cm^−2^ prior to thermal treatment and 1.10 ± 0.05 mg cm^−2^ after annealing, with the minor decrease being attributable to the partial loss of PTFE and volatile organics during the heat treatment step.

The catalytic ink used for dip-coating consisted of 70 wt.% DDM-Ni-P, 20 wt.% PTFE (10 wt.% aqueous dispersion), and 10 wt.% ethanol as the co-solvent. The resulting formulation had a total solid content of 8.5 wt.%, corresponding to a Ni:PTFE mass ratio of 3.5:1, which ensured adequate film cohesion while maintaining sufficient hydrophobic microdomain formation for bubble management. This formulation was optimized to achieve uniform coating, stable binder distribution, and controlled porosity upon drying.

These parameters were explicitly reported to enable precise replication of the catalyst preparation protocol.

### 3.2. The “Zero-Gap” Electrolysis Cell

**Figure 18:**
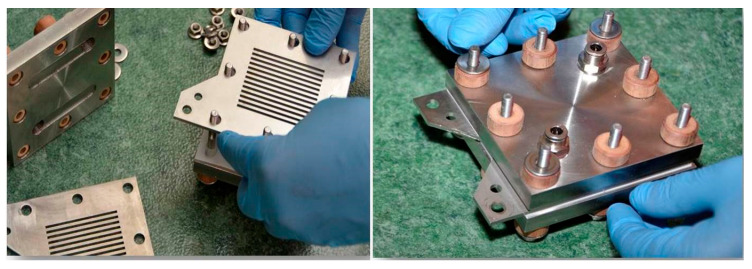
Laboratory “zero-gap” advanced alkaline water electrolysis cell.

The performance of the fabricated electrodes in advanced alkaline water electrolysis is investigated using a laboratory-made “zero-gap” electrolysis cell [65] (Figure 18). It is designed on a modulus principle and can work with up to 12 single cells. In the current research, a one-cell configuration is used. The housing of the cell is made of stainless steel. The electrodes are disk-shaped with a working area of 7 cm^2^. The developed electrodes DDM-NF-CAT and DDM-NF-CAT-A are tested as anodes (oxygen electrode) against an NF-AW cathode (hydrogen electrode). Both electrodes are mechanically attached to a Zirfon Pearl 500 UTP^®^ diaphragm. For assembling the cell, eight stainless steel M6 bolts are used, and uniform compression is ensured by applying torque with a torque wrench. The maximum tightening torque is 12 Nm and is increased stepwise in increments of 2 Nm. After assembly, the cell is tested for leak tightness at 2 bar.

Stainless steel plates that are 50% filled with a 1 mm flow field are used as current collectors and electrolyte distributors. The heating of the cell is ensured using two 48 W heating cartridges (7 cm long) attached onto the current collector plates. The temperature is controlled using a Pt100 thermocouple. The cell is fed with an electrolyte through inlets at the bottom of the anode and cathode compartments, while the corresponding outlets (for the produced gases and the circulating electrolyte) are located at the top.

The Zirfon^®^ membranes are widely applied in advanced alkaline water electrolysis (AWE) since they possess high bubble point pressure (2 ± 1 bar), low ohmic resistance (<0.3 Ω cm^2^), and stability in 30 wt.% KOH solutions at approximately 80 °C. The herein used Zirfon Pearl 500 UTP^®^ membrane consists of an open-mesh polyphenylene sulfide fabric coated with a mixture of 15 wt.% polysulfone and 85 wt.% zirconium oxide [67]. After assembling of the cell (before the electrolysis tests), the membrane is doped with 25% KOH by constant circulation of the electrolyte from the external tank for 24 h at room temperature.

### 3.3. Test Procedure

#### 3.3.1. Physical Characterization

Structural phase analysis of the materials under study is carried out on a Philips ADP15 diffractometer (XRD) (Philips Analytical, Eindhoven, The Netherlands) with using Cu Kα radiation (λ = 0.154 nm) at a constant rate of 0.20·s^−1^ over an angle range 2θ = 10–900.

A JEM-200CX,) scanning electron microscope (SEM) (JEOL Ltd., Tokyo, Japan) is used to examine surface morphology. The presence of iron (VG Scientific, East Sussex, England) is checked by X-ray photoelectron spectroscopy (XPS) on ESCALAB MK II twin anode X-ray source using Mg (hν = 1253.6 eV) and Al Kɑ (hν = 486.6 eV) radiations (VG Scientific Ltd., East Grinstead, UK).

BET surface area, pore size distributions, and total pore volume of the samples are measured using Autosorb iQ (Quantachrome Instruments, Boynton Beach, FL, USA) that has capability of measuring both the adsorbed and desorbed volumes of nitrogen at relative pressures in the range of 0.001 to slightly less than 1.0.

#### 3.3.2. Electrochemical Tests

The initial electrochemical characterization of the synthesized catalyst and the developed DDM-FN-CAT electrode is conducted at room temperature in a three-electrode electrochemical cell (in size of 1 cm^2^) using reversible hydrogen (RHE) reference electrode (HydroFlex and Pt-wire a as counter electrode). The RHE was checked prior to every measurement with the Ag/AgCl electrode in the HCl solution with pH = 0 at 25 °C and it should be equal to 0.244 V.

Unless stated otherwise, all current densities reported in this work are normalized to the geometric surface area of the nickel foam, as determination of the true electrochemically active surface area (ECSA) is complicated by hierarchical porosity and PTFE-induced hydrophobic domains.

The measurements are performed in argon-saturated 1 M KOH aqueous solution at room temperature. The conventional electrochemical techniques of cycling voltammetry and quasi-steady state polarization curves are applied. The cyclic voltammograms (CVs) are recorded in the potential range between hydrogen and oxygen evolution at a scan rate of 100 mV·s^−1^. The quasi-steady state polarization tests (referred to linear sweep voltammetry−LSV) of OER and HER are carried out in a potentiodynamic mode with a scan rate of 1 mV·s^−1^.

It should be noted that all polarization curves presented in this work were collected without applying iR compensation. As a result, the measured potentials include the contribution of the uncompensated solution resistance (R_s_), which is inherent to the electrolyte conductivity, electrode spacing, and contact resistances within the zero-gap assembly. This uncompensated resistance increases the apparent overpotentials in both HER and OER regions, particularly at higher current densities where the iR drop (i × R_s_) becomes significant. Consequently, the extracted Tafel slopes also reflect the combined influence of intrinsic charge-transfer kinetics and ohmic limitations, rather than representing purely kinetic parameters. Therefore, the absolute cell voltages and kinetic values reported here should be interpreted as uncorrected potentials, which are suitable for comparative evaluation of the DDM-modified electrodes under identical conditions, but not as intrinsic catalytic benchmarks.

The electrochemical performance in the “zero-gap” laboratory electrolyzer is examined by chronoamperometry at cell voltages in the range of 1.2 to 2.4 V. These measurements are performed at 20 °C, 40 °C, 60 °C, and 80 °C. The stability of the cell performance is examined under galvanostatic polarization at 1 A cm^−2^ for 1 h at 80 °C. The tested cell operates at atmospheric pressure with zero differential pressure between the anode and cathode compartments. 

## 4. Conclusions

A 3D nickel foam electrode was successfully engineered using a simple and scalable “dip-and-drying” (DDM) method and subsequently coated with a microporous Ni–Fe/PTFE catalytic layer composed of inexpensive and abundant materials (DDM-NF-CAT). Post-annealing (DDM-NF-CAT-A) induced controlled changes in surface wettability, grain morphology, and microcrack formation, which collectively enhanced electrolyte accessibility and moderately improved electrochemical activity. The electrode achieved current densities of 0.325 A·cm^−2^ at 2.0 V and 0.223 A·cm^−2^ at −0.6 V (vs. RHE) in 1 M KOH, and delivered a cell voltage of 2.45 V at 1 A·cm^−2^ and 80 °C in a laboratory zero-gap electrolysis cell. Short-term durability testing at 0.5 A·cm^−2^ over 80 h demonstrated stable operation, confirming the structural robustness of the modified electrode architecture.

Although the measured performance remains modest relative to state-of-the-art zero-gap systems, the primary significance of this work lies in establishing the DDM as a straightforward, reproducible, and scalable approach for the functional modification of nickel foam electrodes. These results provide a proof-of-concept framework for rational electrode design, rather than an immediate industrially deployable solution.

Despite these encouraging findings, several limitations of the current study should be acknowledged. Durability tests were restricted to 80 h at 0.5 A·cm^−2^, primarily at room temperature, which is far below the >1000 h typically required for industrial alkaline water electrolysis validation. The electrode performance remains moderate, indicating that catalyst loading, microstructure, and surface wettability require systematic optimization. Furthermore, no operando diagnostics, such as electrochemical impedance spectroscopy, bubble imaging, or in situ spectroscopic monitoring, were performed to quantify mass-transport effects or track catalyst evolution. Finally, the optimization of key parameters—including annealing temperature, catalyst ink composition, Fe content, and PTFE fraction—was not exhaustive.

Future research will focus on addressing these limitations by optimizing Fe/Ni ratios and PTFE distribution to achieve a balanced surface hydrophilicity and hydrophobicity, conducting long-term stability tests under high-current and elevated-temperature conditions, implementing operando diagnostic techniques to elucidate transport limitations, and exploring alternative binders and post-treatment strategies to reduce overpotentials. Collectively, these efforts are expected to advance the DDM approach toward the development of high-performance, durable electrodes suitable for practical alkaline water electrolysis applications.

## Figures and Tables

**Table 1:** Nickel-based zero-gap alkaline electrolyzers reported in the literature and the present work.

Study/Reference	Separator/Cell Type	Electrode Types	Electrolyte	Anode	Cathode	Temp (°C)	Current Density (A cm^−2^)	Cell Voltage (V)/Notes
Schalenbach et al. (2016) [58]	Zero-gap Zirfon PERL	Ni-based porous electrodes	30% KOH	NiAlMo	NiAl	80	1.0	1.90
Koj et al. (2019) [62]	Zirfon-based zero-gap cell	Ni/NiO electrodes	32.5% KOH	NiFe-GDE	Ni mesh	80	0.4	~2.0
Zayat et al. (2020) [63]	Stainless steel-based cell	SS-based electrodes	30% KOH	α-NiOOH	Ni–Mo	70	0.1	1.71
Záchenská et al. (2022) [65]	Zirfon zero-gap cell	Ni-coated electrodes	30% KOH	Ni–W on NF	Nickel Foam (NF)	80	0.16	2.0
De Groot and Vreman (2021) [77]	Zero-gap Zirfon diaphragm	Raney-Ni (proposed)	30% KOH	Raney-Ni	Ni	80	—	Area resistance: 0.23–0.76 Ω·cm^2^
Henkensmeier et al. (2024) [78]	Porous Zirfon-type (thin)	Ni-based non-noble	30% KOH	-	-	80	1.0	1.83
Luo et al. (2024) [79]	V-Zirfon-350 μm (PVA-skin)	Ni-based	30% KOH	Raney-Ni	NiCoMo-LDH	80	1.3	~2.0
**This work**	**Zero-gap Zirfon^®^-based**	**Fe-NiOOH**	**25% KOH**	**Microporous Layer of Fe-NiOOH and PTFE**	Acid-washed NF (NF-AW)	**80**	**1.0**	2.45; stable

**Table 2:** BET analysis of Ni powder and DDM-Ni-P samples.

Parameters	Ni	DDM-Ni-Powder
BET Surface area (m^2^/g)	1.3	1.6
Total pore volume (cm^3^/g)	0.06	0.06
Pore diameter/BJH Adsorption * (nm)	2.1	2.1
Pore diameter/BJH Desorption * (nm)	2.2	1.7
Avarage pore diameter * (nm)	18	16

* for pores < 395 nm (diameter), P/P_o_ = 0.99.

**Table 3:** Quantitative analysis of the elements based on survey XPS spectra of the electrodes before and after annealing.

	Ni at. %	Fe at. %	O at. %	S at. %	C at. %
DDM-NF-CAT	20.02	4.54	38.07	4.87	32.50
DDM-NF-CAT-A	24.04	2.72	38.10	2.81	32.33

**Table 4:** NF-AW| Zirfon^®^ Perl 500 UTP|DDM-NF-CAT-A performance parameters at 80 °C.

I (A)	U_cell_ (V)	j (A·cm^−2^)	W’ (W·cm^−2^)	W’’_cell_ (W)
0.7	1.70	0.1	0.170	1.19
1.4	1.85	0.2	0.370	2.59
2.1	1.98	0.3	0.594	4.16
2.8	2.10	0.4	0.840	5.88
3.0	2.19	0.5	0.939	6.57
7.0	2.45	1.0	2.450	17.15

## Data Availability

The data that support the findings of this study are available within the articles.

## Abbreviations

The following abbreviations are used in this manuscript:

AWE	Advanced Alkaline Water Electrolysis
DDM	Dip-and-Drying Method
HER	Hydrogen Evolution Reaction
OER	Oxygen Evolution Reaction
RHE	Reference Hydrogen Electrode
NF-AW	Acid-Washed Nickel Foam
